# Psychological distress in individuals with irritable bowel syndrome: the roles of body image and self-criticism

**DOI:** 10.1080/21642850.2024.2334466

**Published:** 2024-03-28

**Authors:** Shulamit Geller, Sigal Levy, Ronit Avitsur

**Affiliations:** a School of Behavioral Sciences, The Academic College of Tel Aviv-Yaffo, Tel Aviv, Israel; b Statistics Education Unit, The Academic College of Tel Aviv-Yaffo, Tel Aviv, Israel

**Keywords:** Irritable bowel syndrome, body appreciation, body dissatisfaction, self-criticism, psychological distress, mediation model

## Abstract

**Objective:**

Irritable bowel syndrome (IBS) is a prevalent gastrointestinal disorder associated with reduced quality of life and psychological distress (PD) including anxiety and depression. The mechanisms linking IBS to PD are unclear. Previous studies showed that body image and self-criticism may be contributory factors. Thus, the objective of this study was to test the potential mediating roles of these factors in the relationship between IBS and PD.

**Method:**

507 adults participated, including 142 with IBS (Mean age = 31.9, SD = 11.7), and 365 healthy peers (Mean age = 26.2, SD = 6.4), ranging in age from 18 to 75. The majority of participants were women (78%). Self-report measures assessed IBS status, body appreciation, body dissatisfaction, self-criticism, sociodemographic status, depression, and anxiety. Path analysis tested the hypothesized mediation model.

**Results:**

IBS participants reported greater PD, lower body appreciation, higher body dissatisfaction, and higher self-criticism than controls. Body appreciation and self-criticism sequentially mediated the link between IBS status and both depression and anxiety. IBS was associated with reduced body appreciation, which in turn was linked to heightened self-criticism, thereby leading to elevated psychological distress.

**Conclusion:**

Results suggest IBS negatively impacts body image appreciation, fostering self-critical judgments that exacerbate psychological symptoms. This study is the first to demonstrate a significant association between body appreciation and IBS, specifically highlighting this relationship. Findings clarify the psychosocial pathways at play in the comorbidity of mental health issues in IBS. Physicians and other health professionals are advised to detect women with IBS who are distressed, and to offer them appropriate intervention to prevent downstream consequences.

## Introduction

Irritable Bowel Syndrome (IBS) is a chronic, debilitating gastrointestinal disorder, characterized mainly by chronic or recurrent abdominal pain and changes in bowel habits (Adriani et al., 2018). IBS is a prevalent condition; studies indicate that it affects 9-23% of the general population, with a predominance among women (80%). IBS results from complex interactions between biological, psychological, and social factors, including food intolerance, enteric infections, dysbiosis, and various psychosocial stressors, thus diagnosis and treatment are challenging (Adriani et al., 2018; Madva et al., 2023). For example, the academic stress was recently demonstrated to be associated with an increase in the prevalence of IBS (Gravina et al., 2023). IBS is a functional disorder, as the diagnosis is based on identification of symptoms reported by the patient while excluding an underling organic disease (Adriani et al., 2018).

While physical symptoms of IBS may be similar to other gastrointestinal conditions, the emotional and psychological impact on patients is distinct and often more difficult than the actual physical discomfort (Houghton et al., 2016). Individuals with IBS report a sense of frustration, associated with a lack of sufficient explanation of their illness by their health-care providers. These feelings may be interpreted as a denial of the legitimacy of their symptoms and lack of empathy (Drossman et al., 2009; Houghton et al., 2016). Patients also report being bothered by the unpredictability of their symptoms and the resulting loss of freedom, spontaneity, and social contacts. The lack of reliability over bodily control and bowel habits further fuels stigma, as family and friends often fail to grasp the validity of symptoms. This perceived judgment from others ties into feelings of fear, shame, and embarrassment common among those with IBS (Drossman et al., 2009; Houghton et al., 2016).

Studies have shown a significant comorbidity between IBS and psychiatric disorders, as 54% to 94% of IBS patients meet criteria for at least one primary psychiatric disorder. Similar to other chronic medical conditions, the most common diagnostic categories are anxiety, depression, and somatization disorders (Khan & Chang, 2010; Whitehead et al., 2002). Furthermore, psychiatric comorbidity was associated with reduced quality of life, exacerbation of IBS symptomatology, and heighten visceral hypersensitivity (Madva et al., 2023). Based on these findings it was suggested that psychological factors play a role in the pathophysiology of IBS, via brain-gut interactions, although reports also support a role for IBS in producing PD symptoms (Schaper & Stengel, 2022). Despite the acknowledged contribution of psychological factors to IBS, the interplay between physiological and behavioral factors in IBS, and the pathways between IBS symptoms and psychological distress (PD), including depression and anxiety, are still unclear.

The current study aims to enhance our understanding of the interactions between IBS and the distress it causes by examining various psychological factors, specifically body appreciation and self-criticism. This approach is critical because understanding these psychological elements can encourage the development of more personalized and effective interventions for individuals suffering from IBS.

Recent research, including studies by Alleva and Tylka (2021), has increasingly focused on the impact of chronic illness and pain on body image, revealing complex interactions between physical health and body perception. Body image is recognized as a multidimensional construct consisting of an individual’s positive and negative perceptions and attitudes toward their body and appearance (Cash, 2004). Historically, the emphasis was on body dissatisfaction or discontent with one's appearance (Fiske et al., 2014; Tiggemann, 2004). However, more recent studies have introduced the distinct construct of positive body image or body appreciation, expanding the scope of body image research. It should be noted that positive body image is not merely the inverse of negative body image – it’s not just the lack of negative body perceptions (for reviews, see Tylka, 2018, 2019).

Body appreciation is characterized by maintaining positive views of one's body, regardless of its physical appearance, and showing respect for one's body regardless of weight and perceived imperfections. This appreciation also involves a resistance to societal pressure to conform to stereotypical beauty standards, while valuing the body's functionality and health (Tylka & Wood-Barcalow, 2015). Generally, patients diagnosed with IBS are found to have a less favorable body image, frequently experience negative bodily sensations, and often perceive their body as a trigger for negative emotions (Bielecka et al., 2021; Jones et al., 2006; Kopczyńska et al., 2018).

Research also shows a strong link between body image concerns and PD in individuals coping with a variety of chronic illnesses, such as fibromyalgia (Berk et al., 2020; Geller et al., 2022), endometriosis (Geller et al., 2021; Sullivan-Myers et al., 2023), IBD (McDermott et al., 2015; Roberts et al., 2022; Wabich et al., 2020), breast cancer (Campbell-Enns & Woodgate, 2015; Trindade et al., 2018), type 1 diabetes (Salah et al., 2022), psoriasis (Gündüz et al., 2020), and systemic lupus erythematosus (Rodrigues et al., 2021). Building on these findings and informed by research that connects body image in IBS with health-related quality of life (Jones et al., 2006; Kopczyńska et al., 2018), we propose that the increased risk of PD in patients with IBS could be partially attributed to a decline in their quality of life due to heightened body dissatisfaction. Moreover, as the concept of positive body image is relatively new, the nature of the relationship between this aspect and PD in individuals with IBS, particularly in the context of reduced body appreciation, is yet to be established.

Elevated negative attitudes and concerns about the body involve undesirable and damaging self-perceptions of being unattractive or worthless (Gilbert, 2002) due to the perceived dissociation from societal body ideals (Quick, 2014; Tiggemann, 2011). These self-evaluations, commonly referred to as self-criticism, can be described as a maladaptive coping strategy consisting of harsh self-scrutiny, perception of the self as inferior or flawed, and an excessive concern with personal failure (for review see Leuke & Skeel, 2017). Self-criticism may be presented as explicit self-critical cognitions, or as feelings of guilt, shame, or anger (Gilbert & Procter, 2006). While some theoretical frameworks, like Gilbert's (2002), indicate a complex and cyclical relationship between body image and self-criticism, we follow Duarte et al.'s (2016) hypothesis that intense self-criticism related to body image mediates the link between chronic illness and symptomatology.

Self-criticism was associated with vulnerability to depression and anxiety in healthy populations, and in people coping with a variety of physical illnesses (Blalock et al., 1995; Kopala-Sibley et al., 2015; Warren et al., 2016). Specifically, in healthy people, self-criticism was a predictor of increased symptoms of depression and anxiety, depressive relapse, and symptoms of residual self-devaluation (Warren et al., 2016), and of a poorer response to anti-depressant treatments (Warren et al., 2016). Within the context of chronic illnesses, self-criticism has been identified as a significant predictor of PD in women with breast cancer (Campos et al., 2012), women managing endometriosis (Geller et al., 2021) and in individuals dealing with conditions such as hypertension, congestive heart failure, rheumatoid arthritis, hyperthyroidism (Pinto-Gouveia et al., 2014), and inflammatory bowel disease (Trindade et al., 2019).

Given the established link between self-criticism and PD in clinical populations, and considering the stigmatizing nature of IBS, we hypothesize that both body image and body appreciation, as well as self-criticism, could be significant factors influencing PD in individuals with IBS.

**Figure 1.**
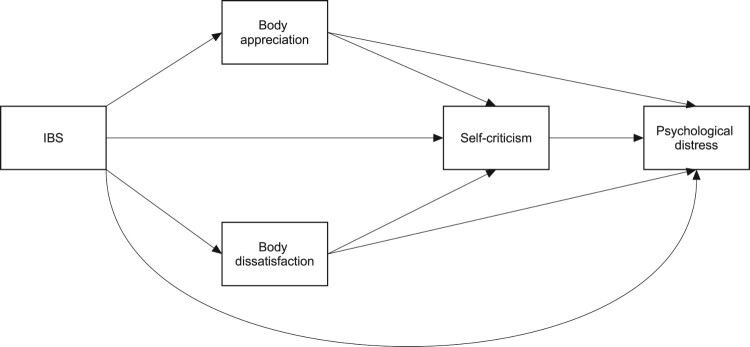
Hypothesized model.

We hypothesize that:

1. Individuals with IBS will have higher levels of depression and anxiety, as compared to their healthy peers.
2. Body appreciation, body dissatisfaction, and self-criticism will mediate the link between IBS and PD such that individuals with IBS will present less body appreciation and higher negative body image. These, in turn, will be related to lower and higher self-criticism, respectively. Self-criticism will be related to higher levels of PD. This hypothesized model is presented in Figure 1.

## Materials and methods

### Participants

The data for this current study was obtained from a cross-sectional survey which was carried out in Israel during 2022–2023. All research was conducted in accordance with the principles of the Declaration of Helsinki and ethics approval was obtained from the ethics committee of Academic College of Tel-Aviv Yaffo (approval code: 2022190 Appendix 1). Participants were recruited via relevant online forums, including those specifically dedicated to supporting individuals coping with IBS, and other general forums. Those who consented to participate in the study received a link to the survey and were requested to complete it electronically. The study group included men and women 20 years of age or older, who self-reported a diagnosis of IBS, and in some cases, other chronic conditions such as hypertension, diabetes, heart conditions, kidney failure, asthma, among others. The control group consisted of participants who reported no existing medical conditions. It should be noted that these diagnoses were not corroborated by medical records.

### Measures

#### Body appreciation

Body appreciation was measured using the Body Appreciation Scale-2 (BAS2) (Tylka & Wood-Barcalow, 2015). This is a 10-item measure that assesses how people accept their body, respect and care for their body, and protect their body from unrealistic beauty standards. Each item ranges from 1 (never) to 5 (always). An overall BAS2 score was computed as the mean of all items, with higher scores indicating greater body appreciation. Internal consistency of the BAS2 in this study was McDonald’s ω = 0.93. This questionnaire was used for patients with IBS and IBD previously (Matos et al., 2021).

#### Body dissatisfaction

Body dissatisfaction was assessed using the Body Dissatisfaction (EDI-BD) subscale of the Eating Disorder Inventory (EDI-2) (Garner, 2004). This 9-item scale assesses dissatisfaction with overall weight and specific areas of the body (e.g. stomach, thighs) ranging from: 1 (always) to 5 (never). An overall EDI-2 score was computed as the sum of all items, with higher scores reflecting greater body dissatisfaction. Internal consistency of the EDI-2 in the current study was sat­isfactory (ω = 0.90). To the best of our knowledge, this questionnaire was not used with IBS patients previously, however it was used for other chronic illnesses (Geller et al., 2021, 2022).

#### Self-criticism

Self-criticism was measured using a 23-item subscale of the Depressive Experiences Questionnaire (DEQ-SC) (Blatt et al., 1976). This subscale reflects concern with failure and with the inability to meet high standards. All items were rated on a 7-point scale ranging from 1 (strongly disagree) to 7 (strongly agree). Scores were obtained by averaging across items, with higher scores indicating greater self-criticism. Internal consistency of the DEQ-SC in this study was McDonald’s ω = 0.91. To the best of our knowledge, this questionnaire was not used with IBS patients previously, however, it was used for other chronic illnesses (Geller et al., 2021, 2022).

#### Depression

Depression was measured using the 9-item Patient Health Questionnaire (PHQ-9) (Kroenke et al., 2001). All items were rated on a 4-point scale ranging from 0 (not at all) to 3 (nearly every day). Total scores were obtained by summarizing the scores of all items. The total score ranges from 0 to 27 with higher scores indicating higher levels of depression. Internal consistency of the PHQ-9 in this study was McDonald’s ω = 0.91. To the best of our knowledge, this questionnaire was not used with IBS patients previously, however it was used for other chronic illnesses (Geller et al., 2021, 2022).

#### Anxiety

Anxiety was measured using the Generalized Anxiety Disorder Scale (GAD-7) (Spitzer et al., 2006). The GAD-7 is a 7-item generalized anxiety measure (panic disorder, social anxiety disorder, and post-traumatic stress disorder). All items were rated on a 4-point scale ranging from 0 (not at all) to 3 (nearly every day). Total scores were obtained by summarizing the scores of all items. The total score ranges from 0 to 21 with higher scores indicating higher levels of anxiety. Internal consistency of the GAD-7 in this study was McDonald’s ω = 0.93. To the best of our knowledge, this questionnaire was not used with IBS patients previously, however, it was used for other chronic illnesses (Geller et al., 2021, 2022).

### Statistical analysis

Descriptive statistics are presented as M(SD) or N(%). The Pearson correlation coefficient was used to assess the association between the continuous variables, and the Chi-Square test was used to assess the association between the categorical one. One-way ANOVA was used to test for group differences in the main study variables. Model 80 of Process v4.2 macro (Hayes, 2018) was used to test the mediation hypothesis. Analysis was done using IBM SPSS v28. Power calculation, done using G*Power 3.1.9.4, affirmed that our sample size provided close to 100% power in all analyses.

## Results

### Study population

Seven hundred and thirty-eight participants accessed the survey link. Of them, 102 were excluded for reporting having a chronic bowel disease that was not IBS (for example, IBD). One hundred and twenty-nine additional participants were excluded for not completing all relevant questionnaires. Thus, the final sample comprised 507 participants. The IBS group included 142 participants, and the majority of this group were women (82%). Control group consisted of 365 healthy individuals of which 77% were women. Sample demographics and group comparison are presented in Table 1.

**Table 1.** Sample demographics and group comparison.

Measure	IBS (N = 142)	Control (N = 365)	Total (N = 507)	F/χ^2^
Age	31.9 (11.7)	26.2 (6.4)	27.8 (8.6)	17.3**
Gender Female Male Other	116 (82) 26 (18)	281 (77) 83 (23) 1 (0.3)	397 (78) 109 (22) 1 (0.2)	1.2
BMI	24.0 (5.2)	22.4 (3.6)	22.8 (4.2)	16.4**
Partnership status In a relationship Not in a relationship	86 (61) 56 (39)	239 (65) 126 (35)	325 (64) 182 (36)	1.1
Age at diagnosis	21.5 (11.2)	NAP		NAP
Age of symptom onset	18.5 (10.6)	NAP		NAP

Note*:* BMI = Body Mass Index, NAP = Not applicable, ** *p *< 0.01

Numbers are M(SD) or N(%). Tests are One-way ANOVA (F statistic) or the Chi-square test (χ^2^ statistic).

The correlations between the main study variables are shown in Table 2, and group comparisons in them are presented in Table 3. All variables are significantly correlated with each other, and there are group differences in all variables. Thus, Hypothesis 1 regarding elevated levels of depression and anxiety among people with IBS was supported.

**Table 2.** Pearson correlations between the main study variables.

	Depression	Anxiety	Body appreciation	Body dissatisfaction
Anxiety	0.72**			
Body appreciation	−0.42**	−0.32**		
Body dissatisfaction	0.25**	0.24**	−0.62**	
Self-criticism	0.54**	0.54**	−0.46**	0.37**

** *p *< 0.01.

**Table 3.** Group comparison in the main study variables.

	IBS (N = 142)	Control (N = 365)	F(1, 505)
Depression	19.1 (6.1)	16.2 (4.9)	29.1**
Anxiety	15.5 (5.8)	14.3 (5.0)	5.4*
Body appreciation	3.2 (0.8)	3.7 (0.7)	34.6**
Body dissatisfaction	2.9 (0.9)	2.7 (0.8)	5.5*
Self-criticism	4.4 (1.0)	4.2 (0.8)	4.8*

* *p *< 0.05; ** *p *< 0.01.

Following the findings presented in Table 4, age and gender were included as covariates in both mediation models, and relationship status was included as a covariate in the model for predicting depression. Due to its known impact on body image, BMI was included as a covariate in both models. Among the IBS patients, no correlations were found between age of diagnosis or onset of symptoms and levels of depression or anxiety.

**Table 4.** Correlations or differences between sample demographics the outcome variables.

Measure	Depression	Anxiety
Age	−0.11*	−0.14**
Gender Female Male	F = 11.0** 17.4 (5.4) 15.5 (5.1)	F = 21.0** 15.1 (5.2) 12.6 (4.6)
BMI	0.08	0.03
Partnership status In a relationship Not in a relationship	F = 9.8** 16.5 (5.0) 18.0 (6.0)	F = 1.9 14.4 (5.1) 15.0 (5.5)
Age at diagnosis	−0.12	−0.02
Age of symptom onset	−0.04	−0.02

Note*:* BMI = Body Mass Index, NAP = Not applicable, * *p < 0.05,* ** *p *< 0.01.

Numbers are Pearson correlations or means (standard deviations).

### Mediation models

#### Depression

**Figure 2.**
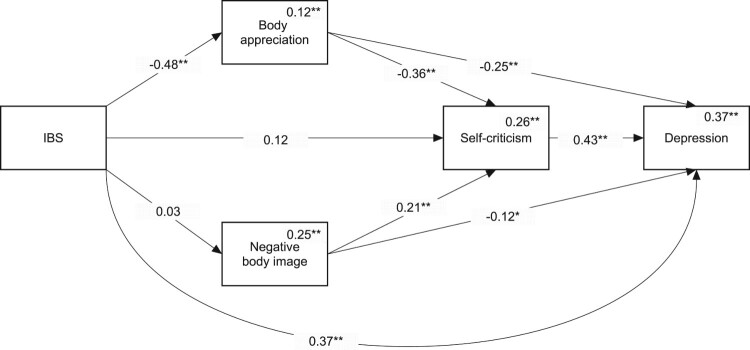
Mediation model predicting depression. Numbers on the lines are standardized path coefficients. Numbers above the variables’ names are multiple squared correlations. While not presented on this graph, age, gender, and BMI were included as covariates in this model. * *p *< 0.05, ** *p *< 0.01.

The mediation model for predicting depression is shown in Figure 2. We found a direct effect of IBS on depression (Beta = 0.37, *p* < 0.01). In addition, we found two indirect paths leading from IBS to depression. One is through body appreciation (Beta = 0.12, 95% CI = [0.06, 0.19]). The other is through body appreciation followed by self-criticism (Beta = 0.08, 95% CI = [0.04, 0.12]). Both indirect paths indicate that people with IBS have a higher level of depression compared to their healthy peers.

#### Anxiety

**Figure 3.**
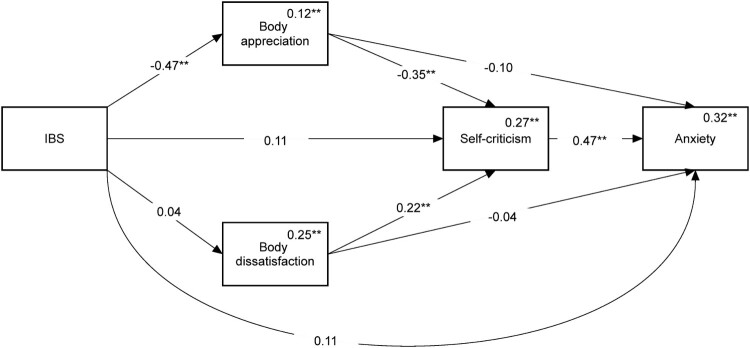
Mediation model predicting anxiety. Numbers on the lines are standardized path coefficients. Numbers above the variables’ names are multiple squared correlations. While not presented on this graph, age, gender, relationship status and BMI were included as covariates in this model. ** *p *< 0.01.

The mediation model for predicting anxiety is shown in Figure 3. No direct effect of IBS on anxiety was found. We found an indirect path leading from IBS to anxiety through body appreciation followed by self-criticism (Beta = 0.08, 95% CI = [0.04, 0.13]).

## Discussion

This study was designed to investigate the factors contributing to the development of PD in individuals dealing with IBS. It specifically focused on understanding how both the positive (body appreciation) and negative (body dissatisfaction) aspects of body image, along with self-criticism, are related to PD in those managing IBS.

The study's findings demonstrate that individuals with IBS reported higher levels of PD, characterized by symptoms of anxiety and depression. This finding is supported by previous reports showing a significant comorbidity between IBS, anxiety, and depression (Khan & Chang, 2010; Whitehead et al., 2002). The psychological effects of IBS are well-documented, with several key factors contributing to increased PD. The unpredictable nature of IBS flare-ups often leads to ongoing worry and impacts daily activities, creating significant stress (Drossman et al., 2009; Houghton et al., 2016). Recurring abdominal pain, a hallmark of IBS, is associated with feelings of helplessness, anxiety, and depression (Franqueiro et al., 2023). Furthermore, the lifestyle restrictions imposed by IBS, such as frequent bathroom trips and dietary changes, can lead to social isolation and loneliness, as individuals may avoid social engagements (Drossman et al., 2009; Houghton et al., 2016). This can result in feelings of embarrassment or stigma, exacerbating social withdrawal and PD (Drossman et al., 2009; Houghton et al., 2016; Islam et al., 2022; Moloney et al., 2016).

Importantly, this study sheds light on body image and self-criticism as critical factors in understanding the connection between IBS and PD. While the influence of body image on PD has been documented in various chronic illnesses (e.g. Geller et al., 2021, 2022), this is the first investigation of its kind in the context of IBS. Moving beyond prior research that primarily concentrated on the negative impact of body dissatisfaction on well-being (Satinsky et al., 2012), our study also explores how the unique concept of body appreciation may be beneficial. Notably, both positive (body appreciation) and negative (dissatisfaction) body image were correlated with PD. However, only body appreciation mediated the link between IBS severity and PD, a relationship similarly observed in the context of other chronic illnesses (Linardon et al., 2022). It thus emphasizes that for individuals with IBS, the root of PD extends beyond mere dissatisfaction with physical appearance (Fiske et al., 2014); it is significantly influenced by the absence of the protective effects that come from valuing one's body (Tylka & Wood-Barcalow, 2015). Consequently, a lower body appreciation in IBS patients contributed to greater self-criticism and PD. Individuals with lower body appreciation are likely to have increased self-judgments regarding their body's functionality, such as perceiving their body as a source of weakness and inaction (Bielecka et al., 2021). This perception can lead to feelings of inadequacy and a lack of trust in their body (Håkanson et al., 2009), which in turn may result in PD. Moreover, the fear of visible bloating or accidental bowel leakage in social situations may promote anxiety around how others perceive them (Drossman et al., 2009). Persistent worry and shame can lead to avoidance, isolation, neglect of bodily care, and increased distress over time (Bielecka et al., 2021). These findings are supported by previous reports highlighting the risk of both lower body appreciation (Trindade et al., 2018), and greater self-criticism in the development of PD across various medical conditions (Geller et al., 2021; Pinto-Gouveia et al., 2014; Trindade et al., 2019). The adoption of a critical and self-deprecating attitude may increase maladaptive defensive responses focused on body image shame (Ferreira et al., 2019). Taken together, the present findings further demonstrate the importance of the interaction between factors such as body appreciation and self-criticism in people coping with chronic illness.

The finding that body appreciation is the sole significant mediator of distress in individuals with IBS is important for multiple reasons. Primarily, it highlights the potential benefits of thoroughly exploring indices of positive body image in relation to IBS. According to body image experts, positive body image is not merely the inverse of negative body image – it’s not just the lack of negative body perceptions (for reviews, see Tylka, 2018, 2019). Thus, considering the relationships between IBS and facets of positive body image – separate from indices of negative body image – will likely enhance our understanding of how body image is associated with IBS. Indeed, prior research (Swami et al., 2020) has shown that body appreciation, as a key component of positive body image, correlates positively with higher quality of life in a range of clinical conditions, such as IBD (e.g. Matos et al., 2021). To our knowledge, however, this study is the first to specifically establish a significant link between body appreciation and IBS.

The study findings indicate that when developing therapeutic interventions for PD in individuals with IBS, emphasis should be placed on promoting body appreciation rather than addressing body image dissatisfaction, while focusing on diminishing self-criticism. This approach could potentially foster greater acceptance of bodily changes and a deeper appreciation beyond conventional beauty standards. This is essential for developing a new identity post-disease and enhancing both quality and meaning of life (Matos et al., 2021).

Alongside these contributions, it is crucial to consider the results of this study in light of its limitations. First, the cross-sectional design employed restricts causal conclusions. Nonetheless, our promising path analytic findings should encourage the design of longitudinal intervention studies that investigate body image, self-criticism, and PD in people with IBS. A second concern is that illness status was reported by the participants and their health status was not directly assessed. An additional examination by a medical professional or screening questions based on ROME IV criteria, would provide direct information for the association between illness and PD. Third, as the study's sample predominantly consisted of females, gathering a more gender-diverse and larger sample is crucial for achieving more reliable conclusions. However, as IBS is more common among females (Adriani et al., 2018; Madva et al., 2023), our sample provides a more solid representation of the IBS population. The fourth limitation is the online recruitment of participants for this study. Previous studies have shown that the method of recruitment may affect the response of women with endometriosis (De Graaff et al., 2015), namely, individuals recruited via support online groups may be those experiencing more severe adverse effects of chronic illness (Armour et al., 2019). This study should therefore be repeated using other recruitment methods. Fifth, limited demographics: as all participants were from a distinct nationality (masked for peer review), findings may not be applicable to other cultural or linguistic groups. Sixth, the severity of IBS symptoms was not measured directly. Gaining insight into the connections between symptom severity and psychological factors would offer further depth to our understanding. Seventh, co-morbidities of other chronic conditions may have affected the results, and this was not examined in the present report. Finally, the study focused specifically on body image and self-criticism variables. Other unaddressed psychological or social mechanisms (e.g. illness perceptions, coping style, social support) may also be relevant in IBS distress.

Given our findings and the study limitations, it is recommended that future research concentrate on creating specialized psychological interventions for those affected by IBS. Focusing on this population, identified as experiencing lower body appreciation and higher self-criticism, could potentially alleviate their symptoms of depression and anxiety, as well as mitigate the adverse effects of IBS, such as shame and diminished self-care. We hope that such studies will contribute to the development of interventions and coping strategies, such as compassionate mind training (CMT) (Gilbert, 2014), or acceptance and commitment therapy (ACT) (Lillis et al., 2009). Focusing on enhancing body acceptance and relieving self-criticism, will likely improve body perception and assist individuals to cope with chronic illnesses.

## Conclusions

Our findings show that people with IBS experience higher levels of depression and anxiety compared to health controls. The innovative aspect of this study lies in demonstrating that issues related to body appreciation and self-criticism could contribute to an elevated risk of psychological distress. In light of our findings, healthcare professionals are encouraged to identify symptoms of anxiety and depression in individuals with IBS and to integrate approaches that facilitate open discussions about body image concerns and promote self-acceptance.

## Authors’ contributions

All authors contributed to the study design and recruitment as well as the composition and revision of this manuscript.

## Institutional Review Board Statement

The study was conducted in accordance with the Declaration of Helsinki and was approved by an Ethics committee. See details under Methods.

## Consent to participate

Informed consent was obtained from all participants in the study.

## Data Availability

The data that support the findings of this study are available on request from the corresponding author. The data are not publicly available due to restrictions, e.g. their containing information that could compromise the privacy of research participants.

## Appendix 1

Ethics Committee Approval – Gastrointestinal Diseases Research Group (IBS, IBD)
